# Immunobiology of Acquired Resistance to Ticks

**DOI:** 10.3389/fimmu.2020.601504

**Published:** 2020-10-14

**Authors:** Hajime Karasuyama, Kensuke Miyake, Soichiro Yoshikawa

**Affiliations:** ^1^ Inflammation, Infection and Immunity Laboratory, TMDU Advanced Research Institute, Tokyo Medical and Dental University (TMDU), Tokyo, Japan; ^2^ Department of Cellular Physiology, Graduate School of Medicine, Dentistry and Pharmaceutical Sciences, Okayama University, Okayama, Japan

**Keywords:** tick-borne diseases, acquired tick resistance, tick saliva antigens, basophil, skin-resident memory T cells, IgE, histamine, epidermal hyperplasia

## Abstract

Ticks are blood-sucking arthropods of great importance in the medical and veterinary fields worldwide. They are considered second only to mosquitos as vectors of pathogenic microorganisms that can cause serious infectious disorders, such as Lyme borreliosis and tick-borne encephalitis. Hard (*Ixodid*) ticks feed on host animals for several days and inject saliva together with pathogens to hosts during blood feeding. Some animal species can acquire resistance to blood-feeding by ticks after a single or repeated tick infestation, resulting in decreased weights and numbers of engorged ticks or the death of ticks in subsequent infestations. Importantly, this acquired tick resistance (ATR) can reduce the risk of pathogen transmission from pathogen-infected ticks to hosts. This is the basis for the development of tick antigen-targeted vaccines to forestall tick infestation and tick-borne diseases. Accumulation of basophils is detected in the tick re-infested skin lesion of animals showing ATR, and the ablation of basophils abolishes ATR in mice and guinea pigs, illustrating the critical role for basophils in the expression of ATR. In this review article, we provide a comprehensive overview of recent advances in our understanding of the cellular and molecular mechanisms responsible for the development and manifestation of ATR, with a particular focus on the role of basophils.

## Introduction

Ticks, especially hard ticks (the *Ixodid* family members), are blood-sucking ectoparasites and serve as vectors for transmission of pathogenic microorganisms, including virus, bacteria and protozoan, that cause serious infectious disorders in animals and humans (1–3). *Ixodid* ticks insert their mouthparts into the host skin and take a blood meal for several days, resulting in increased body weight up to 200-fold. During blood feeding, tick saliva containing a wide range of bioactive substances is injected into host animals to promote successful blood sucking (4–6). During salivation, pathogenic microorganisms can be transmitted from pathogen-infected ticks to host animals. Tick-borne diseases include Lyme disease caused by spirochetes of *Borrelia burgdorferi*, human monocytic ehrlichiosis caused by *Ehrlichia chaffeensis*, Rocky Mountain spotted fever caused by *Rickettsia rickettsii*, virus-mediated encephalitis and sever fever with thrombocytopenia syndrome, and babesiosis caused by protozoa *Babesia* (1–3, 7–9). Apart from tick-transmitted infectious diseases, some people who have experienced tick bites suffer from repeated episodes of systemic anaphylaxis after eating red meat or treated with monoclonal antibodies for cancer therapy. This particular type of allergy is designated as α-gal syndrome, because patients produce IgE against the carbohydrate Galα1-3Galβ1-4GlcNAc-R (α-Gal) that is shared by tick saliva antigens, red meat, and recombinant antibodies (10–12). Thus, tick infestation and tick-borne diseases constitute a growing burden for human and animal health throughout the world.

Most ticks undergo four life stages, namely egg, six-legged larva, eight-legged nymph and adult, taking 2 or 3 years to complete their full life cycle. After hatching, ticks must feed on the blood of host animals at each stage to survive. Most ticks prefer to target a different host animal at each stage. After feeding, larvae and nymphs drop off from hosts and molt to go to the next stage. Not only ticks but also tick-borne pathogens are maintained in this zoonotic cycle. For example, *Ixodes scapularis* larvae and nymphs feed on small rodents such as *Peromyscus leucopus* (white-footed mouse), the main reservoir host for *B. burgdorferi*, a spirochete causing Lyme disease (13). Larvae acquire the pathogen from infected mice and molt to become infected nymphs that in turn feed on other mice, leading to the pathogen transmission to the mice. Infected nymphs molt to become infected adults that feed on white-tailed deer. Although humans are not natural hosts for *Ixodes* ticks, nymphs accidentally feed on humans, resulting in the pathogen transmission to humans and the development of Lyme disease.

For successful blood feeding, ticks inject saliva containing a wide range of bioactive substances into host animals, including vasodilator, anti-hemostatic, anti-inflammatory, and immunosuppressive reagents (4–6). To counteract these, host animals activate various defense pathways, including innate and acquired immunity against tick infestation. Some animal species, including cattle, rabbits, guinea pigs and mice, have been demonstrated to develop resistance to tick feeding after a single or repeated infestation, depending on the combination of tick species and animal species/strains (14–16). This acquired tick resistance (ATR) is manifested by reduced weights of feeding ticks, reduced numbers of engorged ticks, prolonged duration of feeding, inhibition of molting, death of feeding ticks, diminished production of ova or reduced viability of ova. The expression of ATR is not confined to the skin lesion of previous tick bites and can be induced in uninfested skin of sensitized animals, suggesting the involvement of systemic rather than localized responses. ATR was abolished when guinea pigs were treated with immunosuppressants such as methotrexate and cyclophosphamide (17, 18). Furthermore, ATR can be adoptively transferred to naive syngeneic animals with leukocytes or sera isolated from animals infested previously with ticks (19–22). These findings strongly suggested that ATR is a type of immune reaction. From a clinical point of view, ATR is notable, because it can reduce the risk of pathogen transmission from infected ticks to humans and animals (23–26). Hence, further clarification of mechanism underlying ATR will pave the way for the development of efficient anti-tick vaccines to prevent tick infestation and tick-borne diseases.

**Figure 1:**
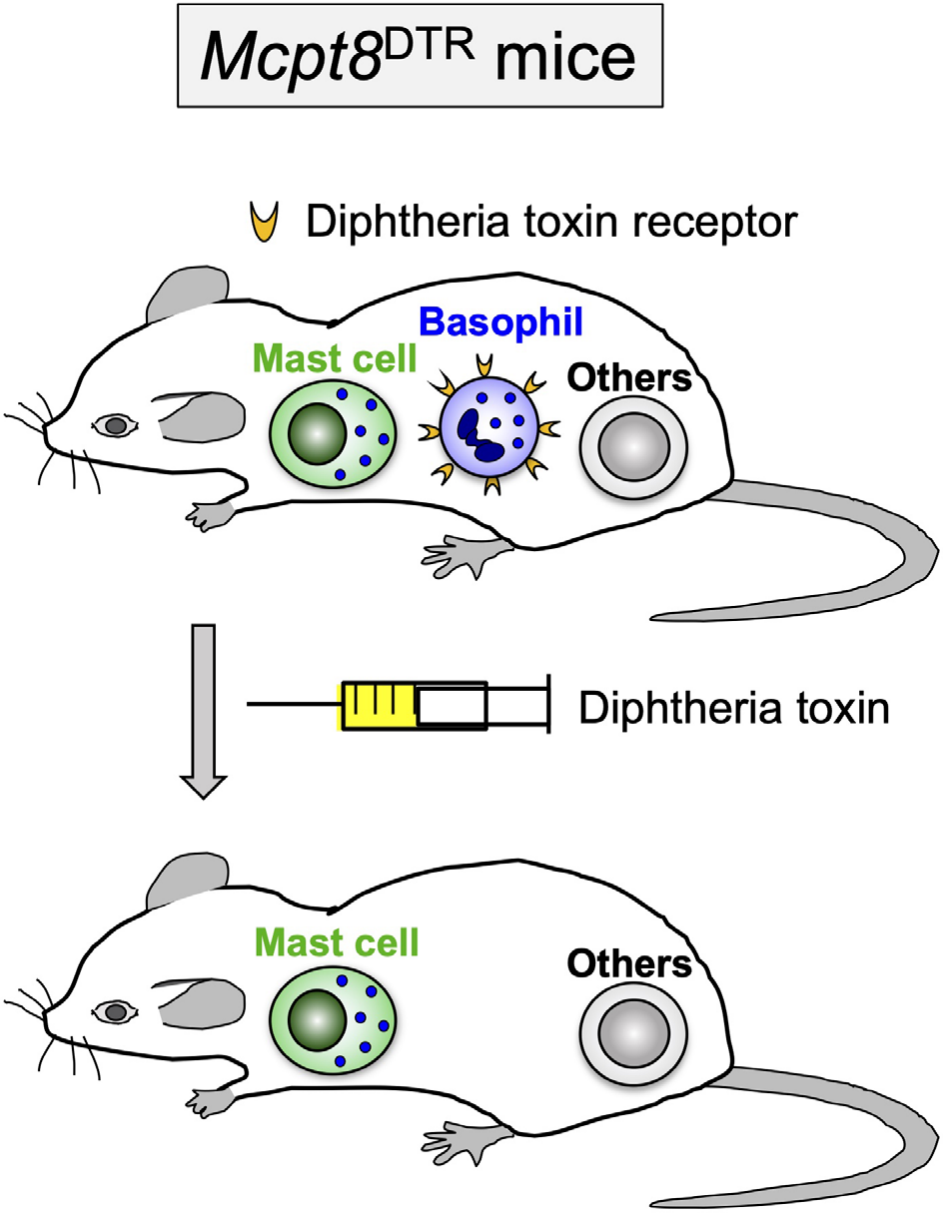
Diphtheria toxin-mediated, conditional depletion of basophils in *Mcpt8* ^DTR^ mice. In *Mcpt8* ^DTR^ mice, the human diphtheria toxin receptor is genetically engineered to be expressed only on basophils, and therefore the diphtheria toxin administration can induce selective ablation of basophils.

Basophils are the least abundant type of granulocytes and account for less than 1% of peripheral blood leukocytes (27, 28). They circulate in the bloodstream under homeostatic conditions and infiltrate peripheral tissues when inflammation occurs there. Although basophils are evolutionally conserved in an array of animal species, their functional roles *in vivo* remained a mystery long after their discovery by Paul Ehrlich in 1879. Basophils are named after basophilic granules in the cytoplasm that stain with basic dyes. In addition to the basophilic granules, blood-circulating basophils share certain phenotypic features with tissue-resident mast cells, including the expression of the high-affinity IgE receptor FcεRI on the cell surface and the release of proallergic mediators such as histamine in response to a variety of stimuli (27, 28). Owing to their phenotypic similarity with mast cells and their rarity, basophils had often erroneously been considered as blood-circulating precursors of tissue-resident mast cells or minor and possibly redundant relatives of mast cells, and therefore neglected in immunological studies (29). Recent development of tools useful for functional analysis, including genetically-engineered mice deficient only in basophils (30–36) (**Figure 1**), has successfully illustrated the nonredundant roles of basophils, distinct from those played by mast cells, in a series of immune responses, including protective immunity to parasitic infections, allergic inflammation, autoimmune diseases, and regulation of innate and acquired immunity (37–39). In this article, we focus on the cellular and molecular mechanisms underlying ATR that have been clarified in animal models of tick infestation.

## Basophils Play a Crucial Role in the Effector Phase of ATR

**Figure 2:**
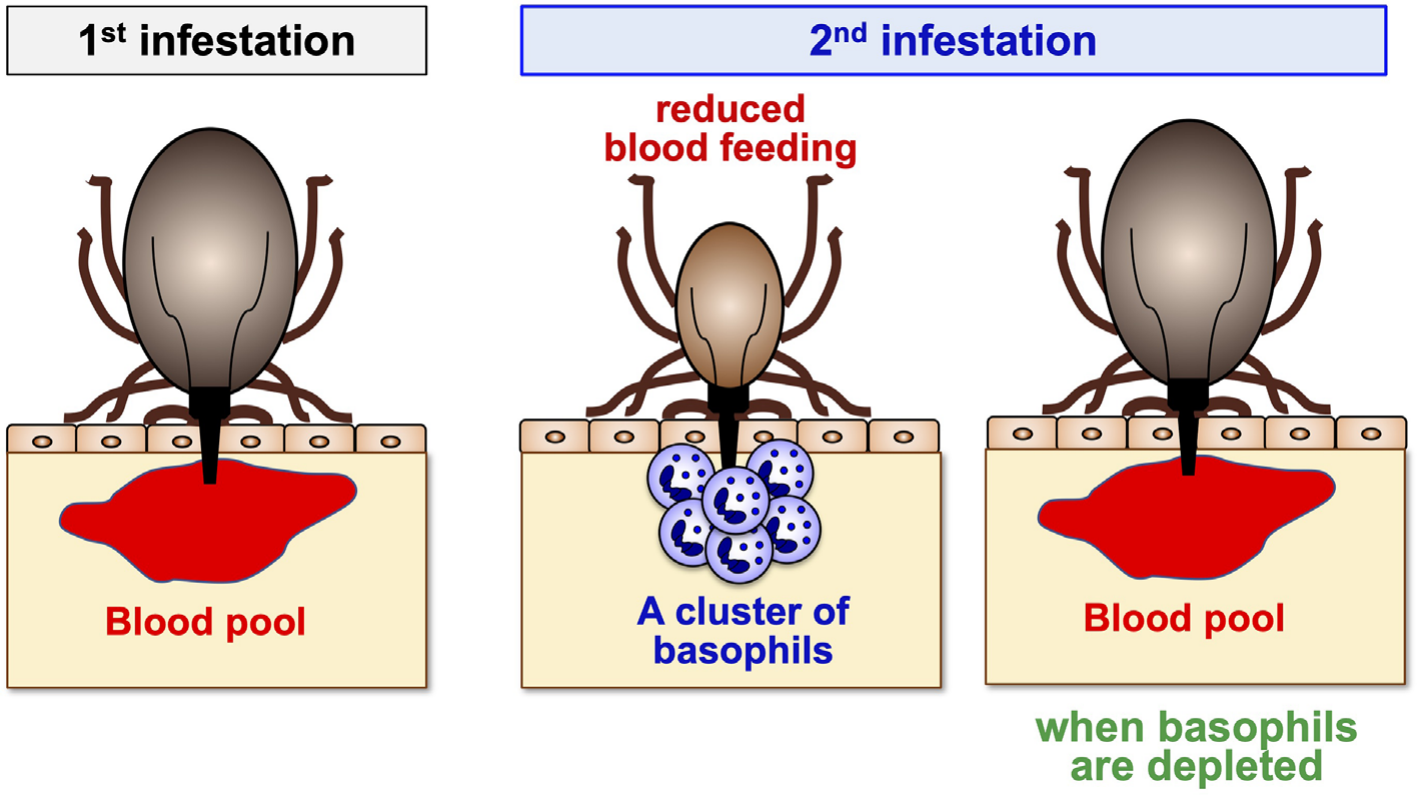
A crucial role for basophils in acquired tick resistance. Some animal species can develop resistance to tick feeding after a single or repeated tick infestation, characterized by reduced body weights of engorged ticks. Basophil accumulation is detected at the tick re-infestation sites of animals showing tick resistance. Basophil depletion just before the 2^nd^ infestation abolishes tick resistance, illustrating a crucial role for basophils in the manifestation of acquired tick resistance.

Trager (14) reported in 1939 that when guinea pigs were repeatedly infested with *Dermacentor variabilis* larval ticks, large numbers of larvae engorged in the 1^st^ infestation whereas relatively few larvae did so in the 2^nd^ or subsequent infestations, indicating guinea pigs developed tick resistance after a single infestation. The resistant state developed within 2 weeks after starting the 1^st^ infestation and lasted for at least 3 months. Skin reaction in tick-resistant guinea pigs was characterized by extensive accumulation of basophils and eosinophils, and basophils composed up to 70% of the skin-infiltrating cells (17). The functional significance of basophil accumulation at the tick-feeding site was illustrated by the depletion of basophils in guinea pigs. Brown et al. (40) established rabbit antiserum against guinea pig basophils, and the treatment of resistant guinea pigs with the anti-basophil serum depleted basophils and abolished ATR, demonstrating a crucial role for basophils in the manifestation of ATR (**Figure 2**). In cattle, rabbits and goats, the infiltration of basophils in the tick re-infestation site was also observed (41–45). The frequency of basophils among cellular infiltrates at the tick-feeding sites varied, depending on the combination of host animals and tick species, and the functional role for basophils of these animals in ATR remains to be investigated.

In mice, conflicting and puzzling findings had been reported, regarding to the contribution of basophils to ATR. Matsuda et al. (46–48) demonstrated that mast cell-deficient WBB6F1-*W/W^v^* mice failed to manifest ATR when re-infested with *Haemaphysalis longicornis* larval ticks, and that ATR was reconstituted by the adoptive transfer of mast cells. Mast cell-sufficient WBB6F1-+/+ congenic control mice showed ATR even though infiltration of basophils was hardly detected histopathologically at the tick-feeding site during the re-infestation. Therefore, it was concluded that mast cells play an essential role in ATR in mice, in contrast to the case of guinea pigs where basophils do so. On the contrary, DenHollander et al. (49) reported that both WBB6F1-*W/W^v^* and WBB6F1-+/+ mice acquired resistance equally well to the infestation with another tick species *D. variabilis* larvae, suggesting that mast cells are dispensable for ATR under these experimental conditions.

It had erroneously been believed for some time that murine basophils either do not exist or are extremely rare. Indeed, it is quite difficult to show their presence in tissue sections by using standard histological methods such as Giemsa stain. The existence of basophils in mice was clearly illustrated by electron microscopic examination (50–52). Steeves et al. (53) used electron microscopy and detected basophils, along with neutrophils and eosinophils, in the tick-feeding skin lesion of both WBB6F1-*W/W^v^* and WBB6F1-+/+ mice infested repeatedly with *D. variabilis* larval ticks, suggesting the possible involvement of basophils rather than mast cells in ATR in mice as observed in guinea pigs. Thus, it remained puzzling whether mast cells and basophils differentially contribute to ATR in mice, depending on different species of ticks, either *H. longicornis* or *D. variabilis*.

Wada et al. (30) addressed this issue and revisited the role of mast cells and basophils in mice repeatedly infested with *H. longicornis* larval ticks. In accordance with the report by Matsuda (46), mast cell-deficient mice failed to show ATR, and the adoptive transfer of mast cells reconstituted ATR, confirming that mast cells are essential for ATR. Moreover, Giemsa staining of skin sections could not detect basophil infiltration at the tick re-infestation sites of mast cell-sufficient mice showing ATR (30), as reported previously (46), suggesting little or no contribution of basophils to ATR. Importantly, however, RT-PCR analysis detected transcripts of the *Mcpt8* gene encoding the basophil-specific protease mMCP-8 in tick-feeding sites during the 2^nd^ but not 1^st^ infestation (30), implying the possible recruitment of basophils to the 2^nd^ tick feeding site. Indeed, histochemical examination of skin sections using the mMCP-8-specific monoclonal antibody demonstrated the accumulation of basophils to the tick-feeding site and their cluster formation surrounding tick mouthparts during the 2^nd^ but rarely the 1^st^ infestation (30) (**Figure 2**). Ohta et al. confirmed this finding by using intravital imaging of *Mcpt8* ^GFP^ mice in that only basophils express green fluorescent protein (GFP) (54). Thus, the recruitment of basophils to the tick re-infested skin lesion was clearly demonstrated in both mice infestated with *H. longicornis* larval ticks (30) and those infested with *D. variabilis* larval ticks (53) as observed in guinea pigs, cattle, rabbits and goats (17, 41–45). Flow cytometric analysis revealed that basophils accounted for less than 5% of leukocytes accumulating at the 2^nd^ tick-feeding site in mice infestated with *H. longicornis* larval ticks, much fewer than in the case of guinea pigs, with monocytes/macrophages, neutrophils and eosinophils being abundant. Nevertheless, basophil ablation by treating mice with basophil-depleting monoclonal antibodies, either anti-CD200R3 (Ba103) or anti-FcεRIα (MAR-1), before the 2^nd^ infestation completely abolished ATR (30) (**Figure 2**). The important role for basophils in ATR was further confirmed by diphtheria toxin-mediated basophil depletion in genetically-engineered *Mcpt8* ^DTR^ mice in that only basophils express diphtheria toxin receptors (30) (**Figures 1** and **2**). Collectively, basophils are key effector cells for ATR in mice infested with *H. longicornis* as reported in guinea pigs. Considering that the accumulation of basophils was also detected in tick re-infestation sites of mast cell-deficient mice that showed ATR to the infestation with *D. variabilis* (49, 53), basophils likely play a crucial role in ATR in mice infested with *D. variabilis* as well. Basophil infiltration was also observed in humans at the tick-feeding sites and the skin lesions of scabies (55–57). Although the role for basophils in ATR has not been demonstrated in humans, it was reported that a patient lacking basophils and eosinophils suffered from widespread scabies (58). This suggests the possible contribution of human basophils to protective immunity against ectoparasites, including ticks.

Mast cells, in addition to basophils, contribute to ATR in mice infested with *H. longicornis* but not with *D. variabilis* (30, 46, 49). It remains to be determined what makes this difference. ATR was completely abolished when either basophils or mast cells were absent in mice infested with *H. longicornis* (30), indicating that the function of basophils and mast cells may not be additive. The accumulation of basophils at the 2^nd^ tick feeding site was normally observed even in mast cell-deficient mice (30), demonstrating that mast cells are dispensable for basophil recruitment. Tabakawa et al. (59) took advantage of intravital imaging using confocal fluorescent microscopy and demonstrated that basophils accumulating in the skin lesion are more motile and make a less dense cluster surrounding a tick mouthpart in the absence of mast cells than in the presence of mast cells. This may suggest that mast cells contribute to ATR by modulating the locomotion of basophils directly or indirectly. The contribution of mast cells to ATR has not been clearly demonstrated in animal species other than mice. The exact role for mast cells in ATR awaits further studies.

## Skin-Resident Memory T Cells Play an Important Role in Basophil Recruitment to the Skin Lesion of Tick Re-Infestation

**Figure 3:**
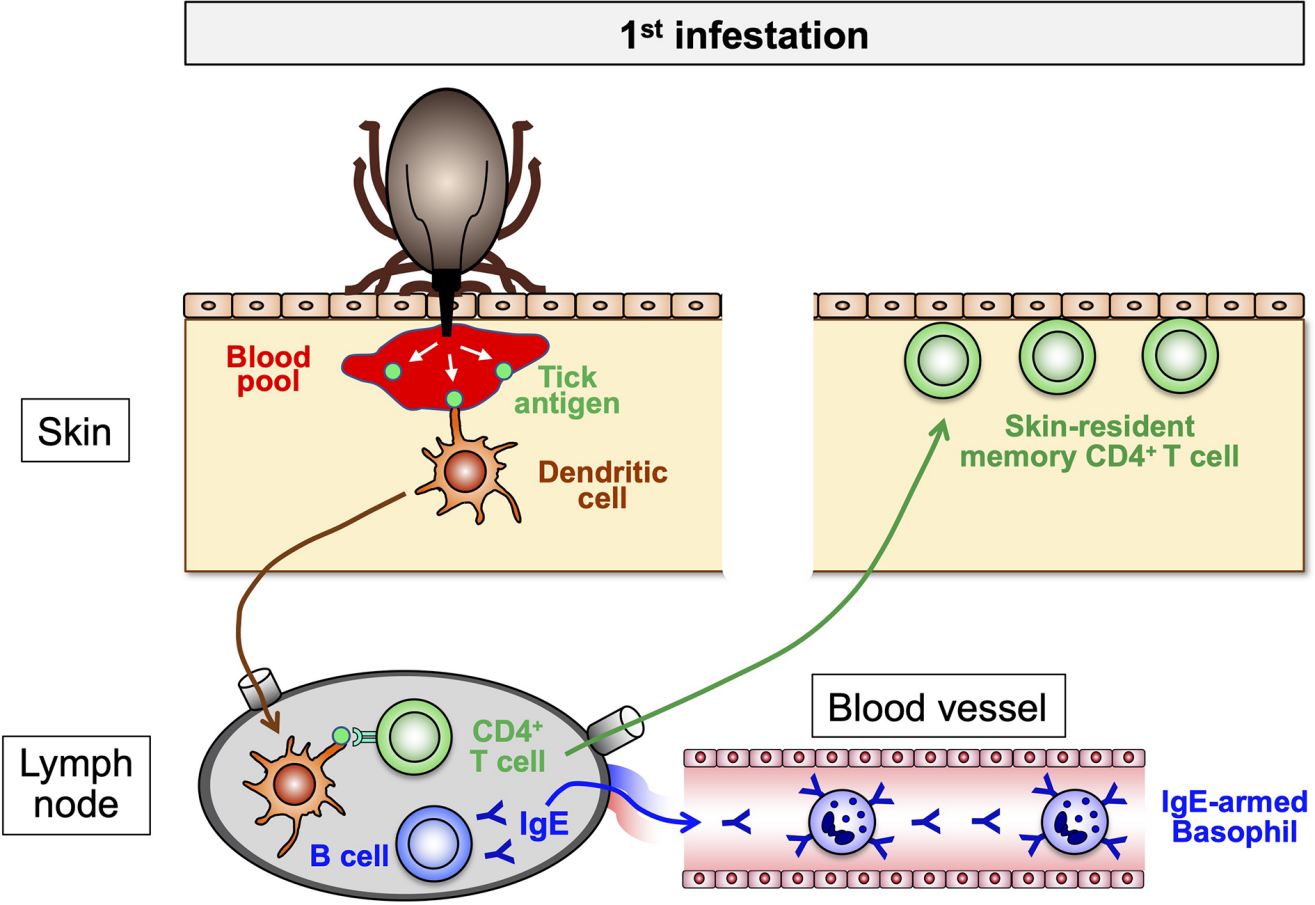
The sensitization phase of acquired tick resistance. In the 1^st^ tick infestation, tick saliva antigens injected into the host skin are taken up by dendritic cells and delivered to draining lymph nodes in that tick antigen-specific B cells and CD4^+^ T cells are activated to expand. The collaboration of these B and T cells promotes the production of tick antigen-specific IgE that enters the blood stream and binds to the surface of blood-circulating basophils through FcεRI. Some of tick antigen-specific CD4^+^ T cells migrate into the skin all over the body and remain as skin-resident, memory T cells.

**Figure 4:**
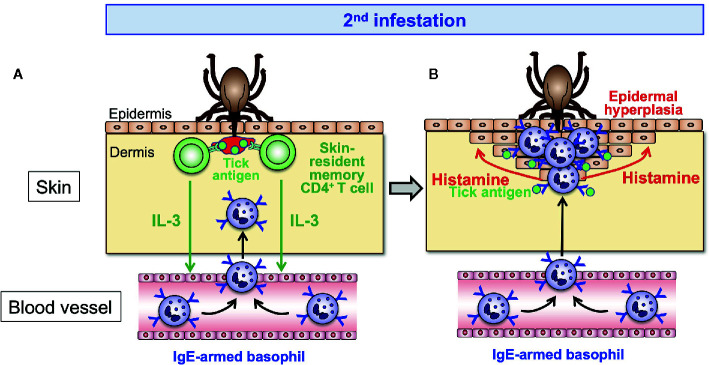
The effector phase of acquired tick resistance. In the 2^nd^ tick infestation, tick saliva antigens injected into the tick-feeding site stimulate the skin-resident, memory CD4^+^ T cells to secrete IL-3 that in turn acts on endothelial cells and promotes transendothelial migration of basophils toward the tick-feeding site **(A)**. Skin-infiltrating basophils make a cluster surrounding the tick mouthpart and are stimulated by tick saliva antigens to release histamine **(B)**. Basophil-derived histamine acts on keratinocytes, leading to epidermal hyperplasia that hampers tick attachment or blood-feeding.

Basophils are not tissue-resident cells and circulate in the blood stream under homeostatic conditions. Basophils infiltrate and accumulate at the tick-feeding sites of some animal species during re-infestation, but hardly during the 1^st^ infestation, to execute ATR (**Figure 2**). Of note, the recruitment of basophils during re-infestation is detected even in previously un-infested skin, distant from the 1^st^ infestation site. This suggests that in response to the 1^st^ infestation, host animals induce some alteration in the skin throughout the body in order to attract basophils to the tick re-infestation site anywhere in the body at any time. Ohta et al. (54) demonstrated in mice infested with *H. longicornis* larval ticks that skin-resident, memory CD4^+^ T cells are critically involved in the recruitment of basophils to the re-infestation site, leading to ATR. In response to the 1^st^ infestation, tick saliva antigen-specific CD4^+^ effector T cells are activated and expand in draining lymph nodes and are distributed to the skin throughout the body, and a fraction of them stay there as skin-resident, memory T cells (**Figure 3**). In the 2^nd^ infestation, tick saliva antigens injected into the tick-feeding site activate these memory T cells to secrete IL-3 that in turn promotes the recruitment of basophils to the tick-infested skin (54) (**Figure 4A**), probably through facilitation of basophil adhesion to endothelium (60–62), leading to transendothelial migration of basophils.

In guinea pigs, complements have been shown to play a part in ATR. Cobra venom factor-mediated depletion of complements blocked ATR in guinea pigs re-infested with *Dermacentor andersoni* larvae, in parallel with reduced numbers of basophils infiltrating the epidermis below the tick attachment site (63). Guinea pigs deficient for the C4 component of complement could acquire and display tick resistance as observed in C4-sufficient guinea pigs (64), suggesting the involvement of the alternative rather than classical pathway of complement activation in ATR. Basophils are chemotactically attracted by fragments of complement components C3 and C5 (65). The deposition of complement components was observed at the dermo-epidermal junction near tick attachment sites and in the basophil-packed epidermal vesicles of resistant guinea pigs (66), suggesting that the complement activation at these sites might contribute to the recruitment of basophils to these sites.

## Tick Saliva Antigens Activate IgE-Armed Basophils *via* FcεRI in the Tick Re-Infestation Site

In both guinea pigs and mice, the transfer of serum from animals previously infested with ticks has been shown to confer ATR on naive animals (20–22, 48), indicating the contribution of tick antigen-specific antibodies to ATR. In mouse experiments, the heat treatment of the serum at 56°C for 2 h abolished its activity of ATR transfer (48), indicating that heat labile IgE antibodies are responsible for the ATR transfer. In accordance with this, both antibody-deficient mice and *Fcer1g* ^-/-^ mice deficient in FcεRI expression showed no ATR (30), suggesting the following steps toward the manifestation of ATR in mice. The 1^st^ tick infestation elicits the production of tick saliva antigen-specific IgE by B cells, and blood-circulating basophils bind such IgE on the cell surface through FcεRI (**Figure 3**). In the 2^nd^ infestation, tick saliva antigens injected into the tick re-infestation site bind to IgE on basophils, resulting in the activation of basophils (**Figure 4B**). Wada et al. (30) demonstrated that both basophils and mast cells critically contribute to ATR in mice infested with *H. longicornis* larval ticks (30), and both of them express FcεRI on the cell surface. Therefore, tick saliva antigens can activate both types of cells through cross-linking the complex of tick antigen-specific IgE and FcεRI on the cell surface. Notably, the adoptive transfer of FcεRI-deficient mast cells conferred ATR on mast cell-deficient mice (30), demonstrating that FcεRI on mast cells is not essential for the manifestation of ATR. In sharp contrast, the adoptive transfer of FcεRI-deficient basophils failed to reconstitute ATR in basophil-depleted mice. Thus, basophils but not mast cells appear to play an important role in IgE-dependent ATR *via* FcεRI-mediated activation in mice, in spite of the fact that both types of cells contribute to ATR. A possible explanation for this will be discussed in the next section.

In guinea pigs, Brown et al. (22) demonstrated that intravenous transfer of immune serum from host animals infested twice with *Amblyomma americanum* larval ticks to naïve animals conferred a significant level of tick resistance. The heat treatment of the serum at 56°C for 4 h had no effect on the serum activity, suggesting little or no contribution of IgE to ATR. The fractionation of the immune serum revealed that IgG1 antibodies are responsible for it. Therefore, IgG1 rather than IgE appears to contribute to ATR in guinea pigs (22).

## Histamine Released From Activated Basophils Promotes Epidermal Hyperplasia, Leading to Tick Resistance

Tick salivary glands contain several histamine-binding proteins, including lipocalins, that are injected into host animals during tick-feeding (4–6). These proteins efficiently compete for histamine with its native receptor such as H1 histamine receptor, implying that histamine produced by host animals could be a threat to successful blood feeding by ticks and therefore must be counteracted. Willadsen et al. (67) reported that the amount of histamine in the skin lesion of individual *Bos taurus* cattle that had received extensive exposure to *Boophilus micropuls* ticks correlates with the degree of resistance to tick infestation. The treatment of cattle with antihistamine lead to higher tick numbers (68) while the injection of histamine into the cattle skin promoted tick detachment (69). Similar observations were reported in guinea pigs infested with *D. andersoni* (70) and rabbits infested with *Ixodes ricinus* (71), suggesting that histamine is an effector molecule involved in ATR in general. Nevertheless, it remained to be determined until recently what types of cells produce histamine and how histamine executes ATR.

Tabakawa et al. (59) addressed these issues by analyzing mice infested with larval *H. longicornis* ticks. In accordance with the previous findings in cattle, rabbits and guinea pigs, the treatment of mice with antihistamine, particularly histamine H1 receptor antagonist, during the 2^nd^ tick infestation abolished ATR. Repeated intradermal administration of histamine or an agonist of histamine H1 receptor beneath the tick-infested site during the 1^st^ infestation significantly reduced the tick feeding as if it were in the second infestation, demonstrating a crucial role of histamine and histamine H1 receptor in the execution of ATR. Wada et al. (30) demonstrated that both basophils and mast cells play key roles in the manifestation of ATR in mice infested with *H. longicornis* larvae, and both types of cells are well-known producers of histamine. Adoptive transfer of histamine-sufficient but not histamine-deficient basophils reconstituted ATR in basophil-depleted mice whereas adoptive transfer of mast cells regardless of histamine sufficiency or deficiency conferred ATR on mast cell-deficient mice (59), indicating that histamine released from basophils but not mast cells is important for the manifestation of ATR. Confocal microscopic examination revealed that basophils accumulated in the epidermis of the 2^nd^ tick-feeding site and formed a cluster that surrounded a tick mouthpart. In contrast, most of mast cells were scattered in the dermis and located away from the tick mouthpart. Considering the fact that histamine has a short half-life, the accumulation of basophils closer to the tick mouthpart, when compared to mast cells, appears to make histamine released from basophils more effective than that from mast cells.

Histamine released from activated basophils appears to contribute to the manifestation of ATR through several different modes of action. Histamine induces itching and grooming responses in the skin, leading to removal of ticks from cattle (72). Paine et al. (73) demonstrated, by using an *in vitro* model of tick feeding through artificial membrane, that the addition of histamine and serotonin to the feeding medium reduced blood feeding and salivation by ticks, suggesting direct effects of histamine on ticks attached to the skin nearby. Epidermal hyperplasia is a characteristic feature at tick-feeding sites of guinea pigs showing ATR (14). Tabakawa et al. (59) reported the epidermal hyperplasia and the cluster of basophils in the thickened epidermis at the 2^nd^ but not 1^st^ tick-feeding site in mice (**Figure 4B**). In this study, the influence of host grooming on tick feeding and skin architecture was negligible, because ticks were confined inside of an acryl ring attached to the skin. Notably, neither histamine-deficient nor basophil-depleted mice showed the epidermal hyperplasia. Repeated administration of histamine beneath the tick infestation site during the 1^st^ infestation induced epidermal hyperplasia, together with the manifestation of tick resistance. These observations suggested that histamine released from basophils is responsible for epidermal hyperplasia. Given that keratinocytes express functional H1 receptor (74) and histamine promotes keratinocyte proliferation (75, 76), it is reasonable to assume that histamine released from activated basophils acts on keratinocytes, leading to the hyperplasia and thickening of epidermis that in turn hamper tick attachment or blood-feeding in the skin during the 2^nd^ infestation (**Figure 4B**).

If the promotion of epidermal hyperplasia by basophil-derived histamine is indeed one of the mechanisms underlying ATR, it is intriguing to hypothesize that the length of tick mouthparts may be correlated with the degree of tick resistance (77). Some ticks such as *H. longicornis*, *D. andersoni*, and *B. microplus* have short mouthparts while others including *A. amricanum* and *Ixodes holocyclus* have longer mouthparts. So, it is plausible that the thickening of epidermis makes the former’s but not the latter’s mouthparts difficult to penetrate deep into the dermis in order to efficiently take a blood meal. In accordance with this assumption, the former tick species are highly responsive to histamine in terms of the induction of tick resistance whereas the latter are less responsive to histamine. Factors other than the length of mouthparts may also influence ATR. For example, the amounts of histamine-binding proteins injected by ticks into host animals may be correlated with differential responsiveness of ticks to histamine in the induction of tick resistance.

## Why and How Do Native Host Animals Show No or Weak ATR in Contrast to Non-Native Hosts?

Many studies on ATR have examined tick feeding on laboratory animals that the particular tick species could not encounter naturally. It is generally thought that when ticks feed on their natural or reservoir host animals, animals show no or weak ATR. In contrast, non-reservoir host animals display strong ATR when repeatedly infested with ticks. For example, *Peromyscus leucopus* (white-footed mouse), the reservoir host for *I. scapularis*, does not show ATR upon repeated infestation with *I. scapularis* nymphal ticks even though they show a strong inflammatory response, including leukocyte accumulation, in the tick-feeding skin lesion (13). This is also the case in laboratory mice (*Mus musculus*) analyzed as surrogates of reservoir host animals. BALB/c and C3H/HeN mice could not develop ATR to nymphal *I. scapularis* or *I. ricinus* ticks upon repeated infestation (78–80). In contrast, these laboratory mice can show strong ATR when repeatedly infested with other tick species, such as *D. variabilis* and *H. longicornis*, that are not native ticks for mice. This suggests that *I. scapularis* has co-evolved with its natural host *Peromyscus leucopus*, and therefore the *I. scapularis*-*P. leucopus* interactions might be optimized for successful tick feeding (81).

The exact mechanism underlying poor development of ATR in natural host animals remains to be determined. Notably, the histopathological comparison of skin lesions of re-infestation with *I. scapularis* nymphal ticks in natural hosts (mice) and non-natural hosts (guinea pigs) revealed that the architecture of the skin lesions was distinct between them even though there was increased inflammation in the dermis of both hosts (13). The tick-feeding site in the non-natural hosts was characterized by a prominent scab-like epidermal hyperplasia and hyperkeratosis whereas the skin structure was not substantially disturbed in the natural hosts. This suggests that a certain step toward ATR development, including the production of tick-specific IgE, the generation of skin-resident, memory CD4 T cells, basophil recruitment, histamine release and epidermal hyperplasia (**Figures 3** and **4**), may not be operative in natural hosts, perhaps due to the modulation of host immune system by tick-derived molecules. Transcriptome and proteome analyses of tick salivary glands demonstrated that ticks of the same species differentially express tick saliva proteins, depending on host animals they feed (80, 82). This difference in the composition of saliva proteins might contribute, in part, to differences in host immune responses. This needs to be taken into account when anti-tick vaccine target antigens are selected. The findings obtained using laboratory animals may not be applied to wildlife animals and humans.

## ATR Can Reduce the Risk of Pathogen Transmission From Infected Ticks to Host Animals

Francis and Little (23) reported that the transmission of *B. bigemina* and *Babesia argentina* to tick-resistant cattle is significantly lower than that to nonresistant hosts. Bell et al. (24) provided clear and convincing evidence that ATR can reduce the risk of pathogen transmission from infected ticks to host animals. Rabbits were infested twice with pathogen-free *D. andersoni*, and they displayed resistance to tick infestation during the 2^nd^ exposure. When infested further with *Francisella tularensis*-infected ticks, only 36% of the tick-resistant rabbits died as a result of *F. tularensis* infection whereas 100% of control naive rabbits died. Nazario et al. (25) demonstrated that repeated infestation of guinea pigs with pathogen-free *I. scapularis* nymphal ticks induced tick resistance in association with reduced capacity of *B. burgdorferi*-infected *I. scapularis* to transmit borrelia infection to guinea pigs. Analysis of people living in Lyme disease-endemic regions demonstrated that residents who experienced itching associated with attached ticks had fewer episodes of Lyme disease than those who reported no such episodes (26). This suggests that acquired immunity to ticks may limit the transmission of *B. burgdorferi* in humans as well.

The exact mechanisms underlying host resistance to tick-borne pathogens in association with resistance to tick infestation remain ill-defined. The resistance to pathogen transmission might be simply ascribed to the decrease of tick feeding and salivation in tick-resistant host animals. However, this does not seem to be always the case. Dai et al. (83) demonstrated that antibodies raised against Salp15, a tick saliva protein, protected C3H/HeJ mice from the transmission of borrelia infection mediated by *B. burgdorferi*-infected *I. scapularis* nymphal ticks. Salp15 binds to the surface of *B. burgdorferi*, increasing the ability of *B. burgdorferi* to infect mice. Salp15 antibodies appear to interact with Salp 15 on the surface of *B. burgdorferi* and hence enhance clearance of spirochete by phagocytes. Salp 15 antibodies showed no apparent influence on the ability of ticks to normally engorge, suggesting that the effect of the antibodies on pathogen transmission cannot be ascribed to the reduced tick feeding/salivation in this case. In accordance with this, an earlier work by Wikel et al. (79) showed that repeated infestation of BALB/c mice with pathogen-free *I. scapularis* nymphal ticks induced host resistance to transmission of *B. burgdorferi* even though mice displayed no apparent ATR. Therefore, the host resistance to tick infestation might not necessarily be a prerequisite for the host resistance to pathogen transmission, even though both types of resistance are often observed in parallel.

## Development of Anti-Tick Vaccines

A number of chemicals have been used for controlling ticks. However, the application of such acaricides has had limited efficacy in reducing tick infestation and often comes with serious side effects, including the selection of acaricide-resistant ticks and the contamination of the environment and animal products, such as milk and meats, with chemical residues. Therefore, alternative strategies for controlling ticks and preventing tick-borne diseases are desired, including vaccines against ticks or pathogens. Because ticks can transmit a variety of pathogens, the development of vaccines against ticks rather than individual pathogens appears to represent one of the most promising and economical alternatives (7, 84, 85). Trager (14) already investigated in 1939 the possibility of the artificial immunization with tick extracts to obtain tick resistance in guinea pigs. Since then, the artificial induction of significant levels of tick resistance has been achieved by immunizing guinea pigs with extracts of tick tissues including salivary glands (21, 86–88). These findings are the basis for the development of tick antigen-based vaccines to forestall tick infestation and tick-borne diseases.

It is important to identify tick salivary antigens that are natural targets of acquired tick immunity, including those critical for ticks to feed, reproduce or transmit pathogens. This helps define salivary protein candidates that can serve as vaccine targets to inhibit tick feeding, reproduction and pathogen transmission to animals and humans. Transcriptomic analyses suggest that ticks of a given species may secrete more than 500 different proteins and peptides in their saliva during blood feeding (89, 90). The composition of saliva appears to change during blood feeding, perhaps confronting the different host defense responses. Targeting salivary proteins expressed early during tick feeding could have the advantage of inhibiting tick feeding early and preventing the pathogen transmission. The functional genomics approach, including RNA interference technology, will help assess the function of each tick gene and identify key molecules that mediate tick-host-pathogen interactions and can serve as vaccine targets (84).

## Conclusions

A series of studies on the cellular and molecular mechanisms underlying ATR suggest the following scenario. In the sensitization phase of ATR (during and after the 1^st^ infestation), tick saliva antigens injected into the skin are taken up by dendritic cells and delivered to draining lymph nodes where tick antigen-specific B and CD4^+^ T cells are activated and expand, leading to the production of tick antigen-specific IgE that in turn binds to the surface of blood-circulating basophils through FcεRI (**Figure 3**). Some of tick antigen-specific CD4^+^ T cells are distributed to the skin all over the body and remain as skin-resident, memory T cells (**Figure 3**). In the effector phase of ATR (during the 2^nd^ infestation), such memory T cells are activated in response to the stimulation with saliva antigens injected by ticks to produce IL-3 that in turn facilitates the recruitment of IgE-armed, blood-circulating basophils to the tick re-infestation site (**Figure 4A**). Skin-infiltrating basophils are stimulated with tick antigens to release histamine that acts on keratinocyte, leading to epidermal hyperplasia that interferes with tick attachment or blood feeding (**Figure 4B**). This is the simplest mode of action proposed for the induction and manifestation of ATR, mainly based on the findings in the models of tick infestation in guinea pigs and mice. Further studies on the tick-host-pathogen interactions are definitely needed to develop the sophisticated strategy to forestall tick infestation and tick-borne diseases.

## Author Contributions

All authors listed have made a substantial, direct and intellectual contribution to the work, and approved it for publication.

## Funding

This work was supported by research grants from Japanese Ministry of Education, Culture, Sports, Science and Technology [19H01025 (HK) and 19K07620 (SY)].

## Conflict of Interest

The authors declare that the research was conducted in the absence of any commercial or financial relationships that could be construed as a potential conflict of interest.
